# Community pharmacists as antimicrobial resistance stewards: a narrative review on their contributions and challenges in low- and middle-income countries

**DOI:** 10.3389/jpps.2024.12721

**Published:** 2024-06-13

**Authors:** Rosy Raju, Sunitha Chandrashekar Srinivas, Srikanth Malavalli Siddalingegowda, Raj Vaidya, Manjiri Gharat, T. M. Pramod Kumar

**Affiliations:** ^1^ JSS College of Pharmacy, JSS Academy of Higher Education and Research, Mysore, India; ^2^ Karuna Trust, Bangalore, India; ^3^ Hindu Pharmacy, Panaji, Goa, India; ^4^ Indian Pharmaceutical Association, Kolkata, India

**Keywords:** community pharmacy, antimicrobial resistance, antimicrobial stewardship, challenges, LMICs

## Abstract

Antimicrobial resistance (AMR) is a global public health crisis that impedes the therapeutic effectiveness of available antimicrobial agents. Due to the high burden of infectious diseases and limited resources, especially trained healthcare professionals, low- and middle-income countries (LMICs) are particularly susceptible to the detrimental effects of AMR. Sometimes, as the first and last point of contact for patients seeking treatment for infections, community pharmacists can play a pivotal role in the stewardship required for AMR. This review aims to highlight the contributions made by community pharmacists in LMICs as AMR stewards. The review considers the challenges from the perspectives of limited resources, inadequate training, a lack of policies and regulations, and issues related to patient behavior. Community pharmacists in LMICs could optimize their advocacy contributions by focusing on One Health AMR stewardship. Transformational and actionable patient and population-centric antimicrobial stewardship (AMS) is feasible with the synergy of policymakers and other healthcare providers in the implementation of AMS policies and programs that support community pharmacists in their efforts to promote rational antimicrobial use.

## Introduction

Antimicrobials have positively impacted global health, saving millions of lives worldwide. They are one of the most prescribed medicines and continue to be increasingly used [1]. Many factors, particularly economic growth, and access to antimicrobials, have contributed to this global increase in consumption [2]. Low- and middle-income countries (LMICs) account for over three-quarters of the increase in antimicrobial consumption. While countries are typically classified based on economic metrics such as gross domestic product (GDP) *per capita*, this category cannot encapsulate a broad spectrum of social, cultural, and healthcare system contexts and differences between them. Many LMICs face at least a double burden of diseases, as they still aim to curb the spread of infectious diseases that have not decreased sufficiently, while simultaneously facing a disproportionate epidemic increase in non-communicable diseases. In the context, the increasing access and usage of antimicrobials has been related to antimicrobial resistance (AMR), an existential threat to public health worldwide [3].

While legal requirements may mandate “prescription-only” access to antimicrobials, not all LMICs are efficient in the stringent implementation of their regulations, resulting in over-the-counter or/- non-prescription dispensing of antimicrobials at retail pharmacies [4]. A systematic analysis identified approximately 4.9 million deaths relating to bacterial AMR in 2019, of which 1.27 million were directly attributable to bacterial AMR [5]. Without appropriate public health interventions, AMR is projected to cause more than 10 million fatalities by 2050, in addition to the accompanying economic losses [6, 7].

LMICs have a higher prevalence of AMR compared to high-income countries (HICs) [8]. Evidence shows that inappropriate antimicrobial usage is common in communities and hospitals in LMICs [9, 10]. Moreover, community pharmacists in LMICs frequently dispense antibiotics without a prescription [11]. One of the main causes of AMR in LMICs is unrestricted access to antimicrobials for self-medication [12]. Community pharmacists in LMICs can play a crucial role in promoting the appropriate use of antimicrobials as frontline healthcare providers. In both rural and urban settings of LMICs, community pharmacies serve as the initial point of contact for patients seeking treatment because they offer readily available and affordable healthcare services. An estimated 80%–90% of human antibiotic use occurs in outpatient settings, contributing to an increasing proportion of resistant infections. Therefore, community pharmacy is an appropriate setting for combating AMR [13]. However, in LMICs, there are many obstacles to addressing AMR in a community setting. Prescribers and community pharmacists cannot fully support antimicrobial stewardship (AMS) efforts due to personal and professional issues such as lack of awareness, fear of losing customers, patient satisfaction and time constraints [14, 15].

The rationale for this review is to explore the possibility of the critical role of AMS among community pharmacists in LMICs. It also aims to highlight the contributions of community pharmacists towards promoting rational antimicrobial use, and educating patients and healthcare providers. Furthermore, it addresses the significant challenges these pharmacists face, such as limited resources, inadequate training, and a lack of stringent policies and regulations, which hinder their efforts in combating AMR effectively. The review emphasizes the necessity for a collaborative approach involving policymakers, healthcare providers, and community pharmacists to optimize the implementation of AMS programs. This aims at enhancing patient and population outcomes while combating the global crisis of AMR.

## Global health and the impact of AMR

The effects of AMR on global health are large and varied. A myriad of negative consequences include a higher incidence of illness, longer hospital stays, higher rates of complications, increased healthcare costs, decreased quality of life for patients, and higher fatality rates [7]. Internationally, an estimate suggests that yearly AMR infections cause over 70, 000 deaths, which will increase disproportionately if the current inaction continues [16]. A further risk to world health is posed by the emergence of infectious strains resistant to antimicrobials. Once-treatable infectious diseases like tuberculosis and pneumonia are evolving with increasing resistance to available antibiotics. This can lead to the spread of incurable infectious illnesses, which is a hazard to global health security [17].

## AMR and its implications for global human health

In addition to the negative health and development consequences, the spread of resistant pathogens can occur across borders, leading to the potential for global pandemics and public health emergencies. People with compromised immune systems, pregnant women, young children, and the older adults are particularly at risk from AMR’s consequences. These individuals are more likely to experience serious complications from infections, making effective treatment difficult. When antibiotics are no longer effective, it can also lead to the use of expensive medicines with lesser safety profiles, compromising patients’ safety and further increasing the burden on patients and healthcare systems. Furthermore, AMR can also have economic implications, as it can lead to decreased productivity while increasing healthcare costs. As more individuals require longer hospital stays and more expensive treatments, it can strain healthcare resources and impact the overall economy due to decreased productivity of people [18].

## Factors contributing to the development and spread of AMR in LMICs

One of the main causes of AMR in LMICs is the excessive and improper use of antibiotics due to over-the-counter availability (OTC). Clinical misuse of antibiotics results from irrational practices of prescribing and dispensing broad-spectrum antibiotics for empiric treatment [13]. Increasingly, viral infection are also frequently treated with antibiotics. Poor infection control procedures at healthcare facilities further aid the emergence and spread of AMR in LMICs [19]. Nosocomial infections in LMICs and inadequate local surveillance are additional factors triggering AMR. This can lead to unnecessary antibiotic use, which could speed up the development of drug-resistant bacterial strains [20]. Further, challenges such as increasing population density, improper solid waste disposal, poor quality of available antibiotics, globalization leading to increased travel, gaps in AMR knowledge, and decreasing vaccination rates for vaccine-preventable diseases, have all catalyzed the spread of AMR. Current concerns are also due to vaccination backlogs during the COVID-19 pandemic, as a result of lockdown and other measures, further exacerbated by vaccine hesitancy and misinformation, resulting in the silent pandemic of AMR [21, 22]. In addition, antimicrobials are inappropriately used in agriculture, being used as growth promoters in the meat, milk, and aquaculture industry among others, resulting in the emergence of bacterial strains that are resistant and transferable to humans [23].

## Challenges faced by community pharmacists in LMICs in tackling AMR

The review of current literature highlights the following challenges faced by community pharmacists in LMICs.

### Limited knowledge

The lack of regular education and training for community pharmacists in LMICs remain a pertinent problem. Lack of continuous professional development (CPD) opportunities in LMIC exacerbates drivers of AMR resulting in antibiotics dispensed without prescriptions. The gaps in community pharmacist’s knowledge limit their capacity to educate patients on rational medication use [24, 25]. Multinational surveys indicates this in Sub-Saharan Africa and Asia [26]. Studies in Benin, India, and Pakistan also show that community pharmacists are unaware of AMR and the rational use of antibiotics [27, 28].

In contrast, mandatory continuing education (CE) requirements keep the United States (US) pharmacists up-to-date on the most recent information on the rational use of antibiotics. Additionally, the latest research in pharmacy practice, the literature on side effect profiles, clinical trials, and newer applications of existing molecules, including off-label use of medicines, and statistics are available to community pharmacists. A few antifungal and antibacterial topical products are available over the counter while antimicrobials are dispensed only based on prescriptions. Pharmacists are also encouraged to review drug utilization, disease correlation, dosage, side effect profile, patient allergy, therapy relevance, and other medications prescribed. These patient-focused activities practiced by the community pharmacists in the United States, among other high income countries (HICs) ensure patient safety and promotes AMS.

### Time constraints

Community pharmacists in LMICs often have a lot of patients due to the increasing population density, which reduces time spent on patient education and adherence. Studies reported from Pakistan, Libya, and Jordan highlight that, pharmacists have challenges taking part in antibiotic awareness campaigns because they lack the time needed [29–31]. The practice of pharmacy will not be altered by any amount of research or statistics unless practicing pharmacists make it a priority to remain informed and are supported to facilitate patient education.

In HICs, though time pressure on community pharmacists is high, supportive mechanisms such as the availability of pharmacy assistants, ready access to medicines information sources, mandatory counselling requirements, and CPD are some of the factors that promote AMS activities. Prioritizing learning remains crucial, resulting in community pharmacists gaining patients’ trust, being well informed and serving their needs. In the USA’s Gallup 2023 Annual Rating of Honesty and Ethics survey, pharmacists were ranked as the third most trusted medical professionals among various occupations. Organizational support, such as that provided by the National Association of Boards of Pharmacy, a nonprofit organization working with the State Boards of Pharmacy, enhances patient and prescription drug safety through pharmacist competency exams, pharmacist license transfer and verification services, and different pharmacy accreditation programs [32].

### Absence of qualified pharmacists

The majority of BRICS countries like Brazil [33], Russia [34], China [35], along with other developing countries like Nigeria, Kenya, Pakistan, and Tanzania are facing shortages of trained pharmacists [36–38]. These challenges significantly limit patient care and lead to problems such as irrational antibiotic use and AMR. A comparative analysis of the latest data on pharmacist density and total pharmacist numbers across several LMICs, according to the WHO, highlights the state of pharmaceutical care infrastructure and its evolution over time (Table 1) [39]. In India, not all registered pharmacists work in retail settings, and those who do are not well compensated.

**TABLE 1:** Availability of pharmacists per 10,000 populations in selected LMICs.

Country	Year	Pharmacists (per 10,000 population)	Total number of pharmacists	Inference
India	2020	8.599	1,200,814	Increased by 6.6 per 10,000 populations, from 2 in 1991 to 8.6 in 2020
Tanzania	2018	0.318	1,845	Increased by 0.29 per 10,000 populations, from 0.031 in 2002 to 0.32 in 2018
Nigeria	2021	0.812	17,331	Increased by 0.35 per 10,000 populations, from 0.46 in 2004 to 0.81 in 2021
Egypt	2018	4.339	45,014	Increased by 1.3 per 10,000 populations, from 3.1 in 2014 to 4.3 in 2018
Jordan	2019	9.847	10,535	Increased by 2.4 per 10,000 populations, from 7.4 in 1998 to 9.8 in 2019
Kenya	2018	0.193	964	Decreased by 0.32 per 10,000 populations, from 0.51 in 2004 to 0.19 in 2018

In addition, there is an increasing number of e-pharmacies in India resulting in increased competition pressuring more community pharmacists to focus on increased sales (which could include dispensing antibiotics without prescription) to make up for the losses, rather than patient services [40]. On the contrary, in the United States, pharmacists must attend CPD sessions periodically, and certified technicians need to renew their certification every 2 years and complete at least 20 h of pharmacy-related continuing education credits (including 1 h of pharmacy law) during that time [41]. These mandated practices and regulations related to rational use of antimicrobials promotes AMS activities in HICs.

### Limited access to updated medicines information

Delivering medicine information to consumer poses significant challenges due to limited access to information for pharmacists in LMICs [42]. However, evidence suggests that drug information centres (DICs) do exist in various countries, including Pakistan [43], Kenya [44], Nigeria [45], Jordan [46], and Nepal [47], which provides services to both healthcare professionals and the public. Additionally, some countries, like the Philippines, have poison information centres alongside DICs [48]. In India, there are approximately 37 DICs, although 250 plus approved Pharm- D colleges which are meant to have DIC services, but these centres often lack the full functionality [49, 50]. In contrast, to obtain authorization to establish a pharmacy in HICs, access to drug reference resources is essential. Hardbound books or electronic drug information resources are widely available and utilized [51]. Thus, the pharmacist is more empowered to cross-refer and validate any prescription-related concerns or discrepancies using the evidence-based sources.

### Patient non-compliance

Intentional or unintentional non-adherence to prescribed and self-medicated antibiotics significantly contributes to the spread of AMR. Furthermore, when patients share unused medications and resort to improper disposal practices, these pose additional challenges for community pharmacists in their efforts to address AMR [52]. Studies conducted in Nigeria [53], Ethiopia [54], India [55], and Bangladesh [56] have shown non-adherence rates to be more than 50%. This is mainly due to factors such as discontinuing treatment when experiencing side effects, feeling better, being unable to afford the full prescription, and preferring to save antibiotics for future use.

### Limited resources

Low profit margins, limited resources, and a poor understanding of how their practices affect public health are some of the challenges or barriers affecting community pharmacists in LMICs as they attempt to address AMS. They may also be under economic pressure from other pharmacies’ competing [57]. In LMICs, community pharmacists must strike a compromise between patient health and business profitability. Community pharmacists sometimes rely heavily on the sale of antibiotics for their revenue, which puts pressure on them to dispense antibiotics that have not been prescribed, including when they are unnecessary. The issue of inadequate funding, support and supervision for AMR-related activities also impedes AMS in community pharmacy settings [58, 59].

### Weak regulatory mechanism

The prevalence of AMR in LMICs is closely linked to the weak regulatory compliance surrounding self-medication with antibiotics. Despite a legal framework in many LMICs that prohibit dispensing antibiotics without a valid medical prescription, OTC sales of antibiotics are frequent [60]. As a consequence of the new and increasing problem posed by AMR; governments worldwide have implemented new National Action Plans (NAPs) to combat it [61]. But in LMICs particularly those without effective regulatory bodies, actions in this direction have been severely lacking in their implementation.

## Strategies in LMICs and HICs to address AMS:

With many obstacles to control the global misuse of antibiotics, many countries have shown success after implementing effective strategies. In many HICs, successful AMS programs incorporating pharmacists have been implemented [62]. The American Society of Health System Pharmacists has identified pharmacists as effective antimicrobial stewards competent in crucial roles within such programs, and a comprehensive approach encompassing surveillance, prevention, stewardship, and innovation has been adopted [63]. In the United Kingdom (UK), the Department of Health and Social Care has developed high-impact interventions utilizing evidence-based tools, such as the *care bundle approach*, aimed to mitigate infection risks. Over the past five to 10 years, the Antimicrobial Stewardship Subgroup has produced numerous guidelines, including the *Target Antibiotics Checklist*, which has helped facilitate improvements in community pharmacy to counsel patients or caregivers being dispensed antibiotics [64].

In LMICs, there is a greater emphasis on AMS approaches within healthcare facilities than in community pharmacies. Examples of such initiatives include, a series of measures implemented to address antibiotic usage, including the introduction of an antibiotic prescription chart, the facilitation of antibiotic stewardship ward rounds led by infection prevention and control specialists, and the restriction of specific antibiotics. These initiatives yielded consistent reductions in overall antibiotic usage and substantial cost savings in South Africa [65]. In Thailand, the *Antibiotic Smart Use* initiative was launched across various healthcare facilities. This comprehensive program engaged local healthcare personnel, policymakers, and researchers to introduce prescriber and patient education, managerial actions promoting herbal medicine for nonbacterial infections, incentives for participation in study visits, and policy adjustments. The program’s success led to its expansion nationwide [66]. Additionally, a study in rural Thailand revealed that implementing a rapid diagnostic test for influenza in outpatients was associated with reduced antibiotic use for negative influenza case [67]. Furthermore; additional initiatives similar to these have been referenced in Table 2.

**TABLE 2:** Highlighted strategies of LMICs to address the local challenges of AMR.

Strategy	Country	Methods	Remedies	Successful outcomes
Roll Back Antimicrobial Resistance Initiative—(AMR School Club) [68]	Tanzania	The establishment of school clubs as a strategy to combat AMR	Students and community members were educated about behaviours that fuels AMR, like hygiene and sanitation, the impact of counterfeit drugs, and a one health approach in classroom sessions	Knowledge was improved from below 37% to above 90%
Improving the management of urinary tract infections (UTIs) in Zambian women using an innovative community engagement approach [69]	Zambia	“*Responsive Dialogues (RD) on Drug Resistance Infections*” framework and toolkit were used in three locations to identify relevant solutions to mitigate AMR in the context of UTIs	RD facilitated dialogue, learning, and solutions to enable change in attitudes, behaviors, policies, and practices regarding the use of antimicrobials	The innovative RD approach helped in breaking some myths and misconceptions about UTIs and UTI management
Facilitating appropriate antibiotic use in respiratory tract infections (RTIs) in children [70]	Kyrgyzstan	The C-reactive protein (CRP) and point of care test (POCT) were used to decide if antibiotics are needed for children with RTIs	A controlled trial to assess the efficacy of a POCT, a qualitative evaluation of its acceptability among healthcare workers and caregivers	A 10% reduction in the unnecessary use of antibiotics among children in the intervention group
Decrease in antibiotic sales in Brazil after new control legislation [71]	Brazil	A copy of the antibiotic prescriptions is to be retained by the pharmacy for audit and there is severe civil and criminal liability if antibiotics are sold without a prescription	Electronic monitoring was used to track information about the antibiotics	Reduction in sales of antibiotics
Impact of point-of- care CRP testing interventions on non-prescription dispensing of antibiotics for RTIs in private community pharmacies [72]	Nigeria	Private community pharmacies (PCPs) in Nigeria that participated were given CRP test kits and additional testing supplies. Staffs were trained in utilizing these kits and interpreting test results to differentiate between viral and bacterial causes in patients with suspected RTIs	CRP testing is used in pharmacies to differentiate between viral and bacterial causes in patients with suspected RTIs	The intervention group significantly reduced the rate of non-prescription antibiotic dispensing by about 16%
Educational intervention on acute respiratory infection management for pharmacies [73]	Bangladesh	Development of ARI guidelines for drug sellers and training on ARI management using developed guidelines at pharmacies drug outlets in each of the 10 zones in Dhaka city, Bangladesh	Drug seller’s response to compliance with guidelines in daily practice	About 99% of drug sellers found the training useful. For children, dispensing of antibiotics for uncomplicated ARI decreased by 9%

## Problem-solving AMS approaches by community pharmacists in LMICs

The role of pharmacists in ensuring the most rational antimicrobial usage is crucial in the fight against the spread of AMR. Following strategies have been incorporated into pharmacy practice in LMICs to tackle AMR.

### Educating patients

Patient-centric counselling with health literacy-appropriate visual aids by community pharmacists that focus on the importance of adherence to prescribed antimicrobials is crucial. One of the key challenges faced by community pharmacists is the lack of knowledge and awareness regarding AMR among patients. Providing comprehensive education and training programs to patients about AMR, appropriate antibiotic use, and infection prevention and control can greatly enhance their understanding of the issue. This facilitates patients adherence to prescribed antibiotics. Utilizing innovative tools such as health literacy-appropriate informational leaflets, posters, and digital platforms can improve patient understanding and promote responsible antibiotic use [74]. Studies have highlighted the importance of medication counseling on how to use antibiotics in LMICs like Ethiopia and Jordan [75, 76]. Additionally, another study in Jordan showed the impact of pharmacist-led education on antibiotic use and resistance through pre- and post-education interventions with patients, demonstrating a significant improvement in participants’ understanding [77].

### Educating school children

Children are often more receptive to learning new concepts and behaviours, and interventions targeted at them have a higher likelihood of success. Instilling important concepts related to AMR, such as proper antibiotic usage, hygiene practices, and understanding the consequences of overuse or misuse of antibiotics, are important disease prevention and health promotion activities. Providing them with appropriate resources facilitates their informed decisions regarding their health and the use of antibiotics, causing the cascading “*triple divident*” that UNICEF has been propagating [78]. When children internalize these lessons, they carry them into adulthood and pass them on to the next-generation, creating a positive cycle of awareness and responsible antibiotic use [79]. A similar example has been applied in Canada “*Do Bugs Need Drugs*” approach to providing educational materials for school children, to raise awareness about antibiotic resistance [80]. By engaging children at a young age, this program and others highlighted in Table 2 aim to instill lifelong habits of responsible antibiotic use, ultimately contributing to the fight against AMR.

### Training both pharmacists and dispensers

In Bangladesh, training programs for pharmacists focusing on Good Pharmacy Practice (GPP) have demonstrated positive outcomes, with trained pharmacists exhibiting better knowledge and practices in dispensing, storage, and customer interaction compared to their untrained peers [81]. Similarly, in Tanzania, brief training for dispensers at Accredited Drug Dispensing Outlets (ADDOs) led to improved treatment practices with antimicrobials and reduced unauthorized dispensing [82]. Studies in Pakistan and the Philippines also showcased significant improvements in dispenser knowledge and practices following training interventions, underscoring the effectiveness of educational initiatives in enhancing pharmaceutical services and patient care [83, 84].

### Promoting infection prevention and control

Community pharmacists possess a unique opportunity to promote public health by raising awareness about practices such as hand hygiene, cough etiquette, vaccination adherence, and staying home when ill. These aspects were well demonstrated in evidence from a survey of community pharmacists in Egypt highlighting good compliance with infection control measures, including mask and glove usage, surface sanitization, and alcohol-based sanitization [85]. The *International Pharmaceutical Federation (FIP)* report on “*What community pharmacy teams need to support good hygiene as part of people’s self-care*” emphasized the importance of pharmacists in India, Indonesia, Saudi Arabia, South Africa, and Thailand, who were involved in public hygiene education [85].

### Collaboration with healthcare providers

Improving patient care requires enhanced collaboration among various healthcare professionals, including community pharmacists. This collaboration involves establishing regular communication channels, exchanging patient information, and promoting interprofessional education. Such collaborations enable pharmacists to actively participate in AMS that contributes to better patient outcomes [86]. A stakeholder engagement process facilitated by the Karnataka State Pharmacy Council in India stated the importance of collaborative approaches and inclusive policy-making in addressing AMS activities at the provincial level [87].

### Supporting surveillance efforts

Establishing robust surveillance systems to monitor antibiotic use and resistance patterns is vital. Community pharmacists can contribute by reporting antibiotic sales, dispensing data, and suspected cases of inappropriate antibiotic use. Integration of community pharmacy data into national surveillance systems when they are functional and well-integrated could enhance the early detection of resistance trends and inform targeted interventions. A study from Japan reported a decrease in community pharmacy sales of certain antibiotics following their AMR Action Plan, highlighting the potential impact of such measures [88]. Similar implementation is required in LMICs. Furthermore, efforts to initiate AMS require collaboration across various stakeholders. The Jordanian Ministry of Health has launched a national antibiotic resistance surveillance system supported by the WHO. Despite collaboration with regulatory bodies like the Jordan Food and Drug Administration (JFDA) and the Jordan Pharmacists Association (JPA), the commitment of healthcare providers, especially community pharmacists, to adhere to these standards remained insufficient [89]. In India, the Revised National Tuberculosis Control Programme (RNTCP) collaborated with the Indian Pharmaceutical Association to release a training module for community pharmacists. These guidelines detail the pharmacist’s role in TB care, including case detection, referral of TB suspects, and rational use of antibiotics and anti-TB drugs, highlighting the crucial role of community pharmacists in combating AMR and enhancing patient care [90].

### Implementing AMS programs:

LMICs are yet to adopt large scale community pharmacist-led AMS initiatives due to inadequate training, a weak regulatory system, and insufficient resources to implement them in their everyday practice. Although pharmacy organizations in LMICs recognize the potential role of pharmacists, actions to address their limitations and competency remain limited [91]. Community pharmacists in LMICs can collaborate with national stakeholders, colleagues, academic institutions, and healthcare providers to implement such programs and promote the responsible use of antimicrobials. One such initiative was observed in India, wherein community pharmacists in Kerala partnered with stakeholders and implemented AMS activities under the Kerala Antimicrobial Resistance Strategic Action Plan (KARSAP). Through a public-private partnership (PPP), they conducted educational outreach and “*Training the Trainers*” sessions to enhance healthcare professional’s capabilities in implementing state antimicrobial policies [92].

### Community pharmacist as a vaccinator

Vaccination continues to be one if the feasible strategies for limiting the spread of vaccine-preventable diseases (VPDs) which further reduces the use of antibiotics and therefore AMR. In HICs, pharmacists are more likely to actively participate in public health initiatives through different methods, including patient counselling, health promotion, and immunizations. VPDs have seen a precipitous decline in prevalence over the past few decades, but this trend has reversed as a result of the COVID-19 pandemic. The inclusion of community pharmacists as vaccinators in LMICs and as vaccine advocates supports public health programs [93]. Such initiatives are observed in a few LMICs, such as the Philippines and Jordan, where pharmacists were trained to administer vaccines [94]. Recently, the Indian Pharmaceutical Association (IPA) and FIP collaborated on a project to training pharmacists to become certified vaccinators and to prepare them to educate their communities [95].

### Compliance with national and international guidelines

In many LMICs, the enforcement of regulations for the rational use of antimicrobials is considered weak, indicating a significant gap between policy and practice. Brazil presents a scenario where stricter regulations are in place, generally prohibiting the dispensation of antibiotics without prescriptions. Similarly, Mexico takes a stricter stance, with antibiotics typically not available over the counter without prescriptions [96]. However, enforcement of these regulations varies, and with some regions experiencing non-compliance [97]. Stringent implementation of policies with the support of regulators is crucial in LMICs to address the “Know-Do” gap in AMS activities.

## Discussion

There is insufficient literature highlighting the role of community pharmacists as antimicrobial stewards, especially in LMICs while broader AMS and One Health AMR initiatives in healthcare facilities and communities have been promoted. These AMS initiatives have the potential to motivate community pharmacists to participate in One Health AMR. However, challenges persist, as evidenced by studies from Ethiopia, South Africa, India, Bangladesh, Nigeria, and Tanzania, which highlight issues such as the inappropriate use of antibiotics and the dispensing of antibiotics without prescriptions, contributing to AMR [98, 99]. Addressing these challenges requires multifaceted approaches, including stringent regulatory frameworks, employee training, and public education campaigns [100, 101].

In addressing specific challenges, various projects are underway to facilitate appropriate antibiotic use in LMICs. For instance, a study in Kyrgyzstan evaluated the effectiveness of a CRP point of care test to reduce unnecessary antibiotic prescriptions for children with respiratory tract infections [69]. Similarly, in Zambia, a community engagement approach is being employed to improve the management of urinary tract infections (UTIs) by fostering awareness and dialogue on AMR issues among stakeholders and communities [68]. Initiatives like employing systematic review methods, participatory workshops, and consultations with experts aimed at strengthening gender equality and social inclusion to address the challenges of AMR, have resulted in the production of user-friendly tools titled *Practical Pathways* in LMICs such as South Africa and Thailand [102].

LMICs can contextualize and adopt programs, such as the UKs Pharmacy Quality Scheme (PQS) to encourage AMS participation, which incentivizes greater engagement in AMS efforts. The Pharmacy Antimicrobial Stewardship Intervention (PAMSI) in the UK could be used to co-develop interventions with pharmacy personnel and stakeholders [103]. This might include community pharmacy-specific tools such as the *Target Antibiotics Checklist* and e-learning sessions. In addition, LMICs can contextualize and use public education campaigns like Canada’s “*Do bugs need drugs?*” focusing on public education about infection prevention through initiatives such as hand washing to stay healthy and stop the spread of the infection, Australia’s Pharmaceutical Society has co-produced continuing education modules on best practice treatment of upper respiratory tract infections and a consumer information brochure and poster on appropriate use of antibiotics [104].

Collaborative efforts between organizations such as the Antimicrobial Resistance and Infection Control (AMRIC) and the Irish Institute of Pharmacy has resulted in developing eLearning resources specific to community pharmacists, which could also be pursued in LMICs [105]. Lastly, LMICs could seek funding for multicomponent interventions such as the *Happy patient project*, focusing on optimizing antibiotic use and reducing AMR through education and feedback mechanisms directly implemented in professional practice settings [106]. These interventions, adapted to local contexts, have the potential to significantly impact AMR in LMICs.

The political momentum to combat AMR is gaining traction globally, with the upcoming United Nations High-Level Meeting scheduled for 26 September 2024, in New York [107]. Organizations such as the International Centre for Antimicrobial Resistance Solutions (ICARS) support the co-creation and co-funding of evidence-based and contextually-appropriate AMR solutions across various sectors [108]. In Tanzania, initiatives such as the Roll Back Antimicrobial Resistance Initiative and the formulation of national action plans demonstrate progress in addressing AMR challenges and educating communities about responsible antibiotic use [109, 110].

Amidst all the challenges that exist in LMICs, the current context has been amplified with additional problems of post-COVID-19 economic problems and climate change consequences, resulting in a drastic loss of momentum for AMR reduction. It is to be noted that AMR and climate change (CC) are two of the top health emergencies, and are interlinked public health priorities because CC will result in outbreaks of zoonotic and vector-borne diseases with pandemic potential [111]. While CC has managed to transition from gaining global attention focused on global as well as local initiatives, AMR is yet to gain the required traction to address a global public health problem. For example, CC has gained suitable political ecology (SPE) by galvanizing scientific basis, mobilization, popularization and polarization; however this is yet to be achieved by AMR [112]. Hence, this requires pharmacists and all health focused associations to leverage the intertwined CC and AMR challenges to bolster the required initiatives for AMR. The current governance systems, such as the G7 and G20 meetings, are also focused on AMR [113], and the One Health approach is being propagated from several sectors, which allows the hardwiring of global and local AMR initiatives to respond to the principles of equity and sustainability.

To address the complex issue of AMR, a comprehensive approach is essential. A project being initiated by the authors of this review, is based on an initial needs assessment at six primary healthcare centres (PHCs) serving tribal and urban poor populations; community pharmacies around these PHCs, and community health workers serving these communities. This project implementation has begun with capacity building of all staff at PHCs, opportunities for training of community pharmacists, and capacity building of community health workers based on their respective health literacy levels. To create sustainable and scalable interventions, these participatory training sessions are focused on strengthening the Medicines and Therapeutics Committees at PHCs. Role-plays based and low-health literate appropriate infographic-based training to strengthen the efforts of community health workers in supporting the Village Health Sanitation and Nutrition Committees (VHSNCs) and Urban Health and Nutrition Days (UHND) is in progress. The challenges of engaging with community pharmacists and unqualified dispensers who are unable to find time for capacity-building exercises continues to remain one of the major challenges of this project.

## Conclusion

Community pharmacists in LMICs face several challenges in initiating AMS, including a lack of education and training, pressure to dispense antibiotics, and weak regulatory enforcement. However, they are in a pivotal position to make significant contributions to addressing this public health challenge. To strengthen their contribution, it is essential to focus on the capacity building of community pharmacists through AMS activities that are well-supported. This also requires stringent implementation of regulatory policies guided by the objectives of the national action plan on AMR, national pharmaceutical policy focused on patient safety and outcomes, and national health policy focused on curative and preventive health.

Collaboration among stakeholders, innovative strategies, and tailored interventions are essential for actionable and sustainable AMS activities. Advocacy and implementation of policies recognizing the role of community pharmacists in AMR stewardship and infection prevention are essential. Task-shifting and capacity building of non-qualified dispensers at the pharmacy based on the level of their health literacy are equally important, so community pharmacists are supported to implement their responsibilities.

Establishing and funding CPD programs focused on updating knowledge and skills related to AMS, rational antibiotic use, and infection prevention are vital. Mandating participation in these programs for LMICs pharmacist registration renewal, as done in HICs, will be a major step towards sustainable competence development. Active engagement by community pharmacists in counseling patients can significantly impact rational antibiotic use and adherence. Lastly, capacity building for additional roles, such as vaccination= advocacy and administration, is crucial. Providing training and certification programs and collaborating with professional associations and regulatory bodies ensures adequate support for community pharmacists involved in such initiatives. Overall, these strategies collectively contribute to initiating AMS and enhancing public health outcomes in LMICs.

## References

[1] Laxminarayan R Duse A Wattal C Zaidi AKM Wertheim HFL Sumpradit N Antibiotic resistance-the need for global solutions. Lancet Infect Dis (2013) 13(12):1057–98. 10.1016/S1473-3099(13)70318-9 24252483

[2] Morgan DJ Okeke IN Laxminarayan R Perencevich EN Weisenberg S. Non-prescription antimicrobial use worldwide: a systematic review. Lancet Infect Dis (2011) 11(9):692–701. 10.1016/S1473-3099(11)70054-8 21659004 PMC3543997

[3] Sifri Z Chokshi A Cennimo D Horng H. Global contributors to antibiotic resistance. J Glob Infect Dis (2019) 11(1):36–42. 10.4103/jgid.jgid_110_18 30814834 PMC6380099

[4] Jacobs TG Robertson J van den Ham HA Iwamoto K Bak Pedersen H Mantel-Teeuwisse AK. Assessing the impact of law enforcement to reduce over the counter (OTC) sales of antibiotics in low- and middle-income countries, a systematic literature review. BMC Health Serv Res (2019) 19(1):536. 10.1186/s12913-019-4359-8 31366363 PMC6670201

[5] Murray CJL Ikuta KS Sharara F Swetschinski L Robles Aguilar G Gray A Global burden of bacterial antimicrobial resistance in 2019: a systematic analysis. The Lancet (2022) 399(10325):629–55. 10.1016/s0140-6736(21)02724-0 PMC8841637 35065702

[6] O’Neill J. Tackling drug-resistant infections globally: final report and recommendations. Review on antimicrobial resistance. Wellcome trust and HM government (2016). Available from: https://amr-review.org/sites/default/files/160525_Final%20paper_with%20cover.pdf (Accessed March, 2023).

[7] Dadgostar P. Antimicrobial resistance: implications and costs. Infect Drug Resist (2019) 12:3903–10. 10.2147/idr.s234610 31908502 PMC6929930

[8] Pierce J Apisarnthanarak A Schellack N Cornistein W Maani AA Adnan S Global antimicrobial stewardship with a focus on low- and middle-income countries: a position statement for the international society for infectious diseases. Int J Infect Dis (2020) 96:621–9. 10.1016/j.ijid.2020.05.126 32505875 PMC7271868

[9] Samreen AI Ahmad I Malak HA Abulreesh HH. Environmental antimicrobial resistance and its drivers: a potential threat to public health. J Glob Antimicrob Resist (2021) 27:101–11. 10.1016/j.jgar.2021.08.001 34454098

[10] Sweileh WM. Global research publications on irrational use of antimicrobials: call for more research to contain antimicrobial resistance. Glob Health (2021) 17:94. 10.1186/s12992-021-00754-9 PMC8383246 34429128

[11] Ahmad T Khan SA Mallhi TH Mannan A Rahman AU Salman M Assessing antibiotic dispensing without prescription through simulated client methodology in developing countries: a comprehensive literature review from 2009 to 2021. J Public Health (2023). 10.1007/s10389-023-02032-x

[12] Barker AK Brown K Ahsan M Sengupta S Safdar N. What drives inappropriate antibiotic dispensing? A mixed-methods study of pharmacy employee perspectives in Haryana, India. BMJ Open (2017) 7(3):e013190. 10.1136/bmjopen-2016-013190 PMC5353334 28255093

[13] Centre for Disease Control. Measuring outpatient antibiotic prescribing. Atlanta, GA, USA: Department of Health and Human Services (2021).

[14] Gebretekle GB Haile Mariam D Abebe W Amogne W Tenna A Fenta TG Opportunities and barriers to implementing antibiotic stewardship in low and middle-income countries: lessons from a mixed-methods study in a tertiary care hospital in Ethiopia. PLoS ONE (2018) 13(12):e0208447. 10.1371/journal.pone.0208447 30571688 PMC6301706

[15] Iskandar K Molinier L Hallit S Sartelli M Hardcastle TC Haque M Surveillance of antimicrobial resistance in low- and middle-income countries: a scattered picture. Antimicrob Resist Infect Control (2021) 10(1):63. 10.1186/s13756-021-00931-w 33789754 PMC8011122

[16] Jee Y Carlson J Rafai E Musonda K Huong TT Daza P Antimicrobial resistance: a threat to global health. Lancet Infect Dis (2018) 18(9):939–40. 10.1016/S1473-3099(18)30471-7 30152350

[17] Salam MA Al-Amin MY Salam MT Pawar JS Akhter N Rabaan AA Antimicrobial resistance: a growing serious threat for global public health. Healthcare (2023) 11(13):1946. 10.3390/healthcare11131946 37444780 PMC10340576

[18] Naylor NR Atun R Zhu N Kulasabanathan K Silva S Chatterjee A Estimating the burden of antimicrobial resistance: a systematic literature review. Antimicrob Resist Infect Control (2018) 7:58. 10.1186/s13756-018-0336-y 29713465 PMC5918775

[19] Leis JA Born KB Theriault G Ostrow O Grill A Johnston KB. Using antibiotics wisely for respiratory tract infection in the era of covid-19. BMJ (2020) 371:m4125. 10.1136/bmj.m4125 33187951

[20] Ayobami O Brinkwirth S Eckmanns T Markwart R. Antibiotic resistance in hospital-acquired ESKAPE-E infections in low-and lower-middle-income countries: a systematic review and meta-analysis. Emerging microbes and infections (2022) 11(1):443–51. 10.1080/22221751.2022.2030196 35034585 PMC8820817

[21] Olorunsaiye CZ Yusuf KK Reinhart K Salihu HM. COVID-19 and child vaccination: a systematic approach to closing the immunization gap. Int J Matern Child Health AIDS (2020) 9(3):381–5. 10.21106/ijma.401 PMC7520883 33014624

[22] Troisi M Andreano E Sala C Kabanova A Rappuoli R. Vaccines as remedy for antimicrobial resistance and emerging infections. Curr Opin Immunol (2020) 65:102–6. 10.1016/j.coi.2020.09.003 33289646

[23] Ibrahim M. Current trends of antimicrobials used in food animals and aquaculture. In: Antibiotics and antimicrobial resistance genes in the environment. Amsterdam, Netherlands: Elsevier (2020). p. 39–69. 10.1016/B978-0-12-818882-8.00004-8

[24] Durand C Chappuis A Douriez E Poulain F Ahmad R Lescure FX Perceptions, current practices, and interventions of community pharmacists regarding antimicrobial stewardship: a qualitative study. J Am Pharm Assoc (2022) 62(4):1239–48.e1. 10.1016/j.japh.2022.02.003 35305926

[25] Klepser ME Dobson EL Pogue JM Labreche MJ Adams AJ Gauthier TP A call to action for outpatient antibiotic stewardship. J Am Pharm Assoc (2017) 57(4):457–63. 10.1016/j.japh.2017.03.013 28499717

[26] Chan AHY Beyene K Tuck C Rutter V Ashiru-Oredope D. Pharmacist beliefs about antimicrobial resistance and impacts on antibiotic supply: a multinational survey. JAC-Antimicrobial Resist (2022) 4(4):dlac062. 10.1093/jacamr/dlac062 PMC9400174 36035318

[27] Allabi A Agbo A Boya B Mudenda S. Antimicrobial stewardship: knowledge and attitudes of pharmacy staff on antibiotic dispensing patterns, use and resistance in Benin. Pharmacol &amp; Pharm (2023) 14:189–214. 10.4236/pp.2023.146014

[28] Tanveer A Kenchey A Mohammed Z Lakshmi PK. Assessment of community pharmacists’ knowledge, attitude and practice on antibiotics and antibiotic resistance. Saudi J Med Pharm Sci (2022) 8(2):92–8. 10.36348/sjmps.2022.v08i02.009

[29] Khan FU Khan FU Hayat K Ahmad T Khan A Chang J Knowledge, attitude, and practice on antibiotics and its resistance: a two-phase mixed-methods online study among Pakistani community pharmacists to promote rational antibiotic use. Int J Environ Res Public Health (2021) 18(3):1320. 10.3390/ijerph18031320 33535695 PMC7908617

[30] Al-Shami HA Abubakar U Hussein MSE Hussin HFA Al-Shami SA. Awareness, practices and perceptions of community pharmacists towards antimicrobial resistance and antimicrobial stewardship in Libya: a cross-sectional study. J Pharm Pol Pract (2023) 16(1):46. 10.1186/s40545-023-00555-y PMC10028782 36945072

[31] Abdel-Qader DH Albassam A Ismael NS El-Shara’ AA Al Meslamani AZ Lewis PJ Community pharmacists’ knowledge of and attitudes toward antibiotic use, resistance, and self-medication in Jordan. Drugs Ther Perspect (2021) 37:44–53. 10.1007/s40267-020-00797-9

[32] National Association of Boards of Pharmacy. Pharmacists ranked third most trusted medical professionals in 2023 Gallup survey - national association of Boards of pharmacy (2024). Available from: https://nabp.pharmacy/news/blog/regulatory_news/pharmacists-ranked-third-trusted-medical-professionals/ (Accessed April 22, 2024).

[33] Al-Worafi YM. Pharmacy education, practice, and research in Brazil. Handbook Med Health Sci Developing Countries (2024) 1–39. 10.1007/978-3-030-74786-2_496-1

[34] Zyukin DA Oleinikova TA Ovod AI Sergeeva NM Reprintseva EV Vlasova OV. The state of the Russian pharmaceutical market from the beginning of the sanctions war to the pandemic. Acad Strateg Manag J (2021) 20:1–9.

[35] Fang Y Yang S Zhou S Jiang M Liu J. Community pharmacy practice in China: past, present and future. Int J Clin Pharm (2013) 35:520–8. 10.1007/s11096-013-9789-5 23661172

[36] Merchant HA Babar ZU Hussain IM. A leap towards enforcing medicines prescribing by generic names in low- and middle-income countries (LMICs): pitfalls, limitations, and recommendations for local drug regulatory agencies. J Pharm Pol Pract (2022) 15(1):104. 10.1186/s40545-022-00501-4 PMC9773520 36550588

[37] Babar ZU. Ten recommendations to improve pharmacy practice in low and middle-income countries (LMICs). J Pharm Pol Pract (2021) 14(1):6. 10.1186/s40545-020-00288-2 PMC7788796 33407945

[38] WHO (World Health Organization). Global health observatory data repository (2023). Available from: https://apps.who.int/gho/data/node.main.HWFGRP_0080?lang=en (Accessed on March 23, 2024).

[39] Sreedhar D Dcruz A Mokashi V Pai S. The rise of E-pharmacy in India: benefits, challenges, and the road ahead. Indian J Pharmacol (2022) 54(4):282–91. 10.4103/ijp.ijp_445_21 36204812 PMC9804119

[40] Alkhateeb FM Shields KM Broedel-Zaugg K Bryan A Snell J. Credentialing of pharmacy technicians in the USA. Int J Pharm Pract (2011) 19(4):219–27. 10.1111/j.2042-7174.2011.00095.x 21733009

[41] Hamid H Masood RA Tariq H Khalid W Rashid MA Munir MU. Current pharmacy practices in low- and middle-income countries; recommendations in response to the COVID-19 pandemic. Drugs Ther Perspect (2020) 36:355–7. 10.1007/s40267-020-00745-7 32837187 PMC7245645

[42] National Drug Use Survey Pakistan. Launched. UNODC (2022). Available from: https://www.unodc.org/pakistan/en/national-drug-use-survey-pakistan-2022-24--launched.html (Accessed April 20, 2024).

[43] Medicines Information. Pharmacy and poisons board (2024). Available from: https://web.pharmacyboardkenya.org/medicines-information (Accessed April 20, 2024).

[44] Okoye I Onyebuchi O. Drug information services utilization in Nigeria from 1980-2020: a narrative review of related studies. J Curr Biomed Res (2022) 2:380–404. 10.54117/jcbr.v2i4.13

[45] Jordan Food and Drug Administration. Jordan food and drug administration (2024). Available from: https://portal.jordan.gov.jo/wps/portal/Home/GovernmentEntities/Ministries/Ministry/Ministry%20of%20Health/Jordan%20Food%20and%20Drug%20Administration/ (Accessed April 22, 2024).

[46] Shrestha S Khatiwada AP Gyawali S Shankar PR Palaian S. Overview, challenges and future prospects of drug information services in Nepal: a reflective commentary. J Multidisciplinary Healthc (2020) 13:287–95. 10.2147/jmdh.s238262 PMC7090186 32256077

[47] UPM. National poison control and information service. Philippines: University of the Philippines Manila (2024).

[48] Chandola AR Kandari S Joshi Y. Status of drug information centre and services in India: an overview and challenges. Int J Pharm Sci Rev Res (2020) 64:60–4. 10.47583/ijpsrr.2020.v64i02.010

[49] Approved institutions for PharmD - PHARMACY COUNCIL OF INDIA. PHARMACY council of India - a statutory body under Ministry of health and family welfare (2023). Available from: https://pcionline.co.in/approved-institutions-for-pharm-d/ (Accessed April 18, 2024).

[50] Borja-Hart NL Leachman BG. Drug information resources used by chain community pharmacists in Tennessee. J Pharm Tech (2016) 32(5):185–90. 10.1177/8755122516653611

[51] Ibrahim MI Assessment of medication dispensing and extended community pharmacy services. InSocial and administrative aspects of pharmacy in low-and middle-income countries. United States: Academic Press (2018). p. 295–309. 10.1016/B978-0-12-811228-1.00018-2

[52] Tobin E Okonofua M Nnadi C Odigie G Osagiede E Atulomah N. Knowledge, prevalence and associated factors for antibiotic non-adherence among adult outpatients in public health facilities in South-south Nigeria. Int J Infect Dis (2020) 101:98. 10.1016/j.ijid.2020.09.278 32916249

[53] Endashaw Hareru H Sisay D Kassaw C Kassa R. Antibiotics non-adherence and its associated factors among households in southern Ethiopia. SAGE Open Med (2022) 10:205031212210904. 10.1177/20503121221090472 PMC9021478 35465633

[54] Bareth H Sharma K Kumar R. Impact of patient counselling on patient adherence with antibiotic drugs: an Indian survey. Pediatrics (2019) 36–53. 10.20959/wjpps20203-15607 30696410

[55] Islam MS Sikdar KY Hossain AMA Faroque AB. Study on the pattern of antibiotic use including the resistance episodes in Bangladesh. Dhaka Univ J Pharm Sci (2019) 18(2):135–43. 10.3329/dujps.v18i2.43255

[56] Chan AHY Darwish R Shamim S Babar Z-U-D. Pharmacy practice and continuing professional development in low and middle-income countries (LMICs). In: Babar Z-U-D, editor. Pharmacy practice. Research case studies. 1st ed. United States: Elsevier Inc. (2021). p. 187–205. 10.1016/B978-0-12-819378-5.00007-6

[57] Bhardwaj K Shenoy M S Baliga S Unnikrishnan B Baliga BS. Knowledge, attitude, and practices related to antibiotic use and resistance among the general public of coastal south Karnataka, India–A cross-sectional survey. Clin Epidemiol Glob Health (2021) 11:100717. 10.1016/j.cegh.2021.100717

[58] Bbosa G Mwebaza N Odda J Kyegombe D Ntale M. Antibiotics/antibacterial drug use, their marketing and promotion during the post-antibiotic golden age and their role in emergence of bacterial resistance. Health (2014) 06:410–25. 10.4236/health.2014.65059

[59] Belachew SA Hall L Erku DA Selvey LA. No prescription? No problem: drivers of non-prescribed sale of antibiotics among community drug retail outlets in low- and middle-income countries: a systematic review of qualitative studies. BMC public health (2021) 21(1):1056. 10.1186/s12889-021-11163-3 34082726 PMC8173982

[60] Heinzel M Koenig-Archibugi M. National action on antimicrobial resistance and the political economy of health care. J Eur Public Pol (2024) 1–27. 10.1080/13501763.2024.2326656

[61] Trivedi KK Dumartin C Gilchrist M Wade P Howard P. Identifying best practices across three countries: hospital antimicrobial stewardship in the United Kingdom, France, and the United States. Clin Infect Dis (2014) 59(Suppl. 3):S170–8. 10.1093/cid/ciu538 25261544

[62] ASHP/SIDP. Joint statement on the pharmacist’s role in antimicrobial stewardship. Am J Health-System Pharm (2023) 81(1), e8. 10.1093/ajhp/zxad275 37949645

[63] Parekh S Hayes CV Loader J Ashiru-Oredope D Hand K Hicks G The use of the TARGET antibiotic checklist to support antimicrobial stewardship in England’s community pharmacies. Antibiotics (2023) 12(4):647. 10.3390/antibiotics12040647 37107009 PMC10135131

[64] Boyles TH Naicker V Rawoot N Raubenheimer PJ Eick B Mendelson M. Sustained reduction in antibiotic consumption in a South African public sector hospital; Four year outcomes from the Groote Schuur Hospital antibiotic stewardship program. South Afr Med J (2017) 107(2):115–8. 10.7196/samj.2017.v107i2.12067 28220735

[65] Sumpradit N Chongtrakul P Anuwong K Pumtong S Kongsomboon K Butdeemee P Antibiotics Smart Use: a workable model for promoting the rational use of medicines in Thailand. Bull World Health Organ (2012) 90:905–13. 10.2471/blt.12.105445 23284196 PMC3524958

[66] Bhavnani D Phatinawin L Chantra S Olsen SJ Simmerman JM. The influence of rapid influenza diagnostic testing on antibiotic prescribing patterns in rural Thailand. Int J Infect Dis (2007) 11:355–9. 10.1016/j.ijid.2006.09.009 17324602

[67] RBA. RBA initiative annual report 2021–2022 (2024). Available from: https://rbainitiative.or.tz/pdf/RBAI-ANNUAL-REPORT-2021-2022.pdf (accessed on March 22, 2024).

[68] ICARS. Improving the management of urinary tract infections in Zambian women through the use of innovative community engagement approaches (2022). Available from: https://icars-global.org/projects/zambia-uti/ (Accessed April 12, 2024).

[69] ICARS. Facilitating appropriate antibiotic use in respiratory tract infections in children in Kyrgyzstan (2024). Available from: https://icars-global.org/projects/kyrgyzstan-rti/ (Accessed on April 12, 2024).

[70] Slain D Lopes-Júnior R De Toledo MI Del Fiol F Barberato-Filho S. Decrease in antibiotic sales in Brazil after new control legislation. Open Forum Infect Dis (2017) 4(1):S61. 10.1093/ofid/ofx162.146

[71] Onwunduba A Ekwunife O Onyilogwu E. Impact of point-of-care C-reactive protein testing intervention on non-prescription dispensing of antibiotics for respiratory tract infections in private community pharmacies in Nigeria: a cluster randomized controlled trial. Int J Infect Dis (2023) 127:137–43. 10.1016/j.ijid.2022.12.006 36509332 PMC9876806

[72] Chowdhury F Sturm-Ramirez K Mamun AA Iuliano AD Chisti MJ Ahmed M Effectiveness of an educational intervention to improve antibiotic dispensing practices for acute respiratory illness among drug sellers in pharmacies, a pilot study in Bangladesh. BMC Health Serv Res (2018) 18:676. 10.1186/s12913-018-3486-y 30170573 PMC6119333

[73] Damisie G Hambisa S Yimam M. Over-the-counter sale of antibiotics at drug stores found in Mizan-Aman Town, Southwest Ethiopia: a cross-sectional simulated client visit study. J pharmaceutics (2019) 2019:1–6. 10.1155/2019/3510659 PMC6475547 31080686

[74] Darwish RM Baqain GN Aladwan H Salamah LM Madi R Masri RMA. Knowledge, attitudes, and practices regarding antibiotic use and resistance among community pharmacists: a cross sectional study in Jordan. Int J Clin Pharm (2021) 43(5):1198–207. 10.1007/s11096-021-01234-1 33515133

[75] Belachew SA Hall L Selvey LA. Community drug retail outlet staff’s knowledge, attitudes and practices towards non-prescription antibiotics use and antibiotic resistance in the Amhara region, Ethiopia with a focus on non-urban towns. Antimicrob Resist Infect Control (2022) 11(1):64. 10.1186/s13756-022-01102-1 35488321 PMC9052473

[76] Shehadeh MB Suaifan GA Hammad EA. Active educational intervention as a tool to improve safe and appropriate use of antibiotics. Saudi Pharm J (2016) 24(5):611–5. 10.1016/j.jsps.2015.03.025 27752235 PMC5059833

[77] Gray NJ Desmond NA Ganapathee DS Beadle S Bundy DA. Breaking down silos between health and education to improve adolescent wellbeing. The BMJ (2022) 379:e067683. 10.1136/bmj-2021-067683

[78] UNICEF. Stepping up effective school health and nutrition: a partnership for healthy learners and brighter futures (2020). Available from: https://www.unicef.org/media/94001/file/Partnership-for-Stepping-up-effective-SHN.pdf.pdf (Accessed March 12, 2024).

[79] Do Bugs. Do bugs need drugs? A community education resource (2024). Available from: https://www.dobugsneeddrugs.org/ (Accessed March 12, 2024).

[80] Ali SB Chakma N Islam MS Amzad R Khan M Aziulla M Assessment of the impact of good pharmacy practices training among drug dispensers in Bangladesh. Front Pharmacol (2023) 14:1139632. 10.3389/fphar.2023.1139632 37502218 PMC10370418

[81] Valimba R Liana J Joshi MP Rutta E Embrey M Bundala M Engaging the private sector to improve antimicrobial use in the community: experience from accredited drug dispensing outlets in Tanzania. J Pharm Pol Pract (2014) 7:11. 10.1186/2052-3211-7-11 PMC4177420 25298887

[82] Hussain A Ibrahim MI Malik M. Impact of educational intervention on knowledge of dispensers working at community pharmacies in Pakistan. Pharm Pract (2013) 11(3):144–8. 10.4321/s1886-36552013000300004 PMC3809132 24223079

[83] Philippine Tuberculosis Initiatives for the Private Sector. Pharmacy-based TB DOTS implementation plan. Manilla (2003). Available from: https://pdf.usaid.gov/pdf_docs/Pnacw833.pdf (Accessed April 11, 2024).

[84] Elsayed AA Darwish SF Zewail MB Mohammed M Saeed H Rabea H. Antibiotic misuse and compliance with infection control measures during COVID-19 pandemic in community pharmacies in Egypt. Int J Clin Pract (2021) 75(6):e14081. 10.1111/ijcp.14081 33559255 PMC7995210

[85] International Pharmaceutical Federation. What community pharmacy teams need to support good hygiene as part of people’s self-care? The Hague (2023). Available from: https://www.fip.org/file/5479 (Accessed April 16, 2024).

[86] Sakeena MHF Bennett AA McLachlan AJ. Enhancing pharmacists’ role in developing countries to overcome the challenge of antimicrobial resistance: a narrative review. Antimicrob Resist Infect Control (2018) 7:63. 10.1186/s13756-018-0351-z 29744044 PMC5930749

[87] Srinivas SC Prasad R George S Satyaseela MP Coetzee R. Stakeholder engagement at Karnataka state pharmacy Council for development of a state-level antimicrobial stewardship policy. SA Pharm J (2020) 87(1):52–4.

[88] Hasegawa K Mori T Asakura T Matsumura Y Nakaminami H. Surveillance of antimicrobial prescriptions in community pharmacies located in Tokyo, Japan. Antibiotics (2023) 12(8):1325. 10.3390/antibiotics12081325 37627745 PMC10451865

[89] Saleh D Abu-Farha R Mukattash TL Barakat M Alefishat E. Views of community pharmacists on antimicrobial resistance and antimicrobial stewardship in Jordan: a qualitative study. Antibiotics (2021) 10(4):384. 10.3390/antibiotics10040384 33916855 PMC8067308

[90] Central TB Division. Ministry of Family Welfare and Indian Pharmaceutical Association revised national tuberculosis control programme training module for community pharmacists (2024). Available from: https://tbcindia.nic.in/WriteReadData/l892s/6538189187Training%20Module%20for%20Community%20Pharmacists.pdf (Accessed April 22, 2024).

[91] Abubakar U Tangiisuran B. Knowledge and practices of community pharmacists towards non-prescription dispensing of antibiotics in Northern Nigeria. Int J Clin Pharm (2020) 42:756–64. 10.1007/s11096-020-01019-y 32270378

[92] Singh S Charani E Devi S Sharma A Edathadathil F Kumar A A road-map for addressing antimicrobial resistance in low-and middle-income countries: lessons learnt from the public private participation and co-designed antimicrobial stewardship programme in the State of Kerala, India. Antimicrob Resist Infect Control (2021) 10:32–9. 10.1186/s13756-020-00873-9 33573697 PMC7878026

[93] Le LM Veettil SK Donaldson D Kategeaw W Hutubessy R Lambach P The impact of pharmacist involvement on immunization uptake and other outcomes: an updated systematic review and meta-analysis. J Am Pharm Assoc (2022) 62(5):1499–513.e16. 10.1016/j.japh.2022.06.008 PMC9448680 35961937

[94] International Pharmaceutical Federation (FIP). Public health emergencies. Federation (2024). Available from: https://www.fip.org/public-health-emergencies#vaccine (Accessed April 22, 2024).

[95] Indian Pharmaceutical Association Community Pharmacy Diviion. 56th IPAA CPD e- Times (2024). Available from: https://ipapharma.org/ipa-cpd-e-times/ (Accessed May 14, 2024).

[96] Santa-Ana-Tellez Y Mantel-Teeuwisse AK Dreser A Leufkens HG Wirtz VJ. Impact of over-the-counter restrictions on antibiotic consumption in Brazil and Mexico. PloS one (2013) 8(10):e75550. 10.1371/journal.pone.0075550 24146761 PMC3797702

[97] Adamu AA Gadanya MA Jalo RI Uthman OA Wiysonge CS. Factors influencing non-prescription sales of antibiotics among patent and proprietary medicine vendors in Kano, Nigeria: a cross-sectional study. Health Policy Plan (2020) 35(7):819–28. 10.1093/heapol/czaa052 32529246

[98] Sulis G Gandra S. Access to antibiotics: not a problem in some LMICs. Lancet Glob Health (2021) 9(5):e561–2. 10.1016/S2214-109X(21)00085-1 33713631

[99] Auta A Hadi MA Oga E Adewuyi EO Abdu-Aguye SN Adeloye D Global access to antibiotics without prescription in community pharmacies: a systematic review and meta-analysis. J Infect (2019) 78(1):8–18. 10.1016/j.jinf.2018.07.001 29981773

[100] Dache A Dona A Ejeso A. Inappropriate use of antibiotics, its reasons and contributing factors among communities of Yirgalem town, Sidama regional state, Ethiopia: a cross-sectional study. SAGE open Med (2021) 9:20503121211042461. 10.1177/20503121211042461 34504704 PMC8422824

[101] Sono TM Markovic-Pekovic V Godman B. Effective programmes to reduce inappropriate dispensing of antibiotics in community pharmacies especially in developing countries. Adv Hum Biol (2024) 14(1):1–4. 10.4103/aihb.aihb_128_23

[102] ICARS. Strengthening gender equality and social inclusion in LMICs across the AMR research continuum; ICARS (2024). Available from: https://icars-global.org/projects/gender-amr/ (Accessed on April 12, 2024).

[103] Hayes CV Parekh S Lecky DM Loader J Triggs-Hodge C Ashiru-Oredope D. The national implementation of a community pharmacy antimicrobial stewardship intervention (PAMSI) through the English pharmacy quality Scheme 2020 to 2022. Antibiotics (2023) 12(4):793. 10.3390/antibiotics12040793 37107155 PMC10135021

[104] International Pharmaceutical Federation (FIP). Fighting antimicrobial resistance: the contribution of pharmacists. The Hague, Netherlands: International Pharmaceutical Federation (2015). (Accessed April 13, 2024).

[105] International Pharmaceutical Federation (FIP). Antimicrobial resistance and stewardship education: supporting the pharmaceutical workforce in AMR and AMS. The Hague, Netherlands: International Pharmaceutical Federation (2023). (Accessed April 13, 2024).

[106] Bjerrum A García-Sangenís A Modena D Córdoba G Bjerrum L Chalkidou A Health alliance for prudent prescribing and yield of antibiotics in a patient-centred perspective (HAPPY PATIENT): a before-and-after intervention and implementation study protocol. BMC Prim Care (2022) 23(1):102. 10.1186/s12875-022-01710-1 35501712 PMC9063370

[107] UN High Level Meeting on AMR: Countdown begins. ReAct (2024). Available from: https://www.reactgroup.org/news-and-views/news-and-opinions/year-2024/un-high-level-meeting-on-amr-countdown-begins (Accessed April 15, 2024).

[108] ICARS. Partnering with LMICs (2024). Available from: https://icars-global.org/partner/partnering-with-lmics/ (Accessed April 18, 2024).

[109] Queen's Commonwealth Trust. RBA Initiative - leading the fight against antimicrobial resistance. Queen's Commonw Trust (2024). (Accessed April 18, 2024).

[110] The United Republic of Tanzania. National action plan 2023-2028. United States: World Health Organization (2023). (Accessed April 22, 2024).

[111] Magnano San Lio R Favara G Maugeri A Barchitta M Agodi A. How antimicrobial resistance is linked to climate change: an overview of two intertwined global challenges. Int J Environ Res Public Health (2023) 20(3):1681. 10.3390/ijerph20031681 36767043 PMC9914631

[112] Zaman AW. The drivers of global attention in complex and creeping crises: the cases of antimicrobial resistance and climate change. Roskilde Universitet. FS and P Ph.D. afhandlinger (2022). Available from: https://rucforsk.ruc.dk/ws/portalfiles/portal/81573852/The_drivers_of_global_attention_in_complex_and_creeping_crises_phd.pdf (Accessed April 23, 2024).

[113] FIF. Recommendations on global health for the 2023 G7 summit (2023). Available from: https://ifi.u-tokyo.ac.jp/en/wp-content/uploads/2023/04/policy_recommendation_tg_20230322e.pdf (Accessed April 23, 2024).

